# Horizontal ridge reconstruction of the anterior maxilla using customized allogeneic bone blocks with a minimally invasive technique - a case series

**DOI:** 10.1186/s12903-017-0423-0

**Published:** 2017-12-08

**Authors:** Laurent Venet, Michel Perriat, Francesco Guido Mangano, Thomas Fortin

**Affiliations:** 1 Department of oral surgery, Hospices Civils de Lyon, Lyon, France; 2 Department of Medicine and Surgery, Dental School, University of Varese, Varese, Italy; 3 Department of Oral Surgery, Dental School of Lyon, University Claude Bernard, Lyon 1, 6-8 rue Guillaume Paradin, 69007 Lyon, France; 4 UJF-Grenoble 1 / CNRS / TIMC-IMAG UMR 5525, F-38041 Grenoble, France

**Keywords:** Allograft, Bone block, Augmentation, Custom made, Stereolithographic model

## Abstract

**Background:**

Different surgical procedures have been proposed to achieve horizontal ridge reconstruction of the anterior maxilla; all these procedures, however, require bone replacement materials to be adapted to the bone defect at the time of implantation, resulting in complex and time-consuming procedures. The purpose of this study was to describe how to use a 3D printed hardcopy model of the maxilla to prepare customized milled bone blocks, to be adapted on the bone defect areas using a minimally invasive subperiosteal tunneling technique.

**Methods:**

Cone beam computed tomography (CBCT) images of the atrophic maxilla of six patients were acquired and modified into 3D reconstruction models. Data were transferred to a 3D printer and solid models were fabricated using autoclavable nylon polyamide. Before the surgery, freeze-dried cortico-cancellous blocks were manually milled and adapted on the 3D printed hardcopy models of the maxillary bone, in order to obtain customized allogeneic bone blocks.

**Results:**

In total, eleven onlay customized allogeneic bone grafts were prepared and implanted in 6 patients, using a minimally invasive subperiosteal tunneling technique. The scaffolds closely matched the shape of the defects: this reduced the operation time and contributed to good healing. The patients did not demonstrate adverse events such as inflammation, dehiscence or flap re-opening during the recovery period; however, one patient experienced scaffold resorption, which was likely caused by uncontrolled motion of the removable provisional prosthesis. Following a 6 month healing period, CBCT was used to assess graft integration, which was followed by insertion of implants into the augmented areas. Prosthetic restorations were placed 4 months later.

**Conclusions:**

These observations suggest that customized bone allografts can be successfully used for horizontal ridge reconstruction of the anterior maxilla: patients demonstrated reduced morbidity and decreased total surgery time. Further studies on a larger sample of patients, with histologic evaluation and longer follow-up are needed to confirm the present observations.

## Background

Bone resorption after tooth loss is progressive and irreversible [1–3]. This may lead to severe hard tissue deficiency requiring bone block grafting prior to placement of an endosseous implant, which is a technique well-recognized in the literature [1–3].

In the anterior maxilla, in particular, bone resorption following the loss of one or more teeth can result in a horizontal bone deficiency; such a deficiency can make the placement of dental implants difficult, also because the insertion of an implant in a thin maxillary alveolar ridge can cause major aesthetic problems [1, 3–5].

Autogenous bone has always been considered the material of choice for horizontal regeneration of the anterior maxilla, through the placement of onlays bone blocks harvested in intra- or extra-oral sites [4–6]; the placement and subsequent integration of these onlays blocks is prerequisite for the correct positioning of dental implants [4–6]. However, the use of autogenous bone has drawbacks, such as limited availability and the need to be harvested from other anatomical sites, with a risk of morbidity at donor site and resorption at recipient site [2, 3, 7, 8]. In addition, harvesting autogenous bone blocks is time-consuming and stressful for the surgeon [2, 3, 7, 8].

In order to overcome the limitations related to the use of autogenous bone, several alternative materials such as allografts [9, 10], xenografts [11] sythetic and composite bone grafts [12, 13] have been proposed.

To date, allografts are often the most preferred alternative to autografts for bone grafting surgeries; in particular, mineralized cortico-cancellous allograft bone can be used for bone regeneration in dentistry, to replace autogenous bone block grafts [9, 10].

Irrespective of used material, key factors to consider for predicting success include perfect adaptation of the block graft to recipient site in order to increase stability, preservation of blood supply by keeping the raised flap in good condition and maintaining edge to edge closure of the surgical site following surgery [14, 15].

To improve fit and reduce surgical time, in a case report published in 2006 Jacotti et al. [16] were the first to adapt a bone block on a three-dimensional (3D) solid jawbone model, to allow practitioners to evaluate fit using various points of view, while eliminating any visual intraoral obstacle. This milled block was transferred into the surgical site after flap elevation and screwed in without additional modifications. Moreover, use of image-guided surgery has also been suggested to reduce the invasiveness of procedures and to minimize surgical trauma, risk of infection, patient discomfort and morbidity [17, 18]. Although the case report published by Jacotti et al. [16] presented a novel and extremely interesting technique, that can be successfully used for the treatment of deficient alveolar ridges, so far there are no clinical studies in the literature on the use of customized allogeneic bone grafts for bone regeneration.

Accordingly, our present study aimed to, 1) describe the use of a 3D printed jawbone model to prepare customized allogeneic bone blocks, and 2) to place the milled blocks on the defect areas with a minimally invasive sub-periosteal tunneling technique, to improve blood supply. The clinical translational goal of this work is to promote correct integration of grafting material serving as a scaffold for new bone reconstruction.

## Methods

### Patient selection

Six consecutive patients with compromised anterior maxillary ridges, who presented for the placement of dental, were included in this case series. All aspects of this study complied with standards of the Declaration of Helsinki (revision of 2008). Prior to participation, all patients provided written informed consent. Inclusion criteria were patients with an age > 18 years, with an horizontal maxillary bone deficiency, with the need of bone grafting prior to implant placement. Exclusion criteria were history of recent infection, pregnancy, metabolic disorders, immunocompromised status, drug or alcohol abuse, history of radiation therapy in the head and neck, psychiatric disorders and inability to understand the described procedure and to sign the informed consent form. Study ethics approval was obtained on 30th may 2014 by CECIC Rhone-Alpes-Auvergne, Clermont Ferrand, IRB 5891.

### Planning procedure

**Fig. 1:**
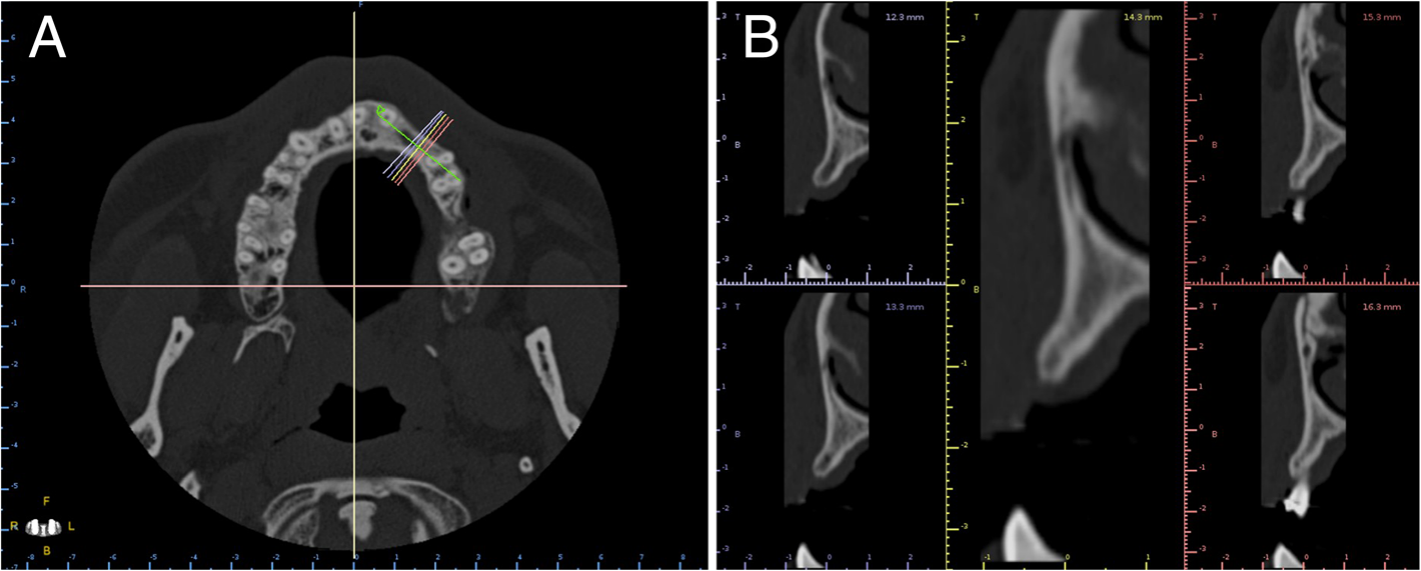
Preoperative anatomic situation on CBCT axial (**a**) and cross-sectional (**b**) images

Primary planning was based on intraoral observations, intraoral radiography and cross-sectional imaging using cone beam computed tomography (CBCT) (Fig. 1a, b). When the dental surgeon decided the appropriate treatment was bone grafting, the patient was informed of the availability of an alternative, conventional treatment procedure involving autologous bone block accompanied by a raised full-flap technique. Following a complete examination of the patient, during the planning stage the restorative clinician created a study prosthesis using diagnostic casts representing the final restorative prosthesis. Following satisfactory testing in the mouth of the patient, the study prosthesis was duplicated in acrylic resin while serving as a scanning template. Teeth were fabricated in radiopaque acrylic resin.

The digital imaging and communication in medicine (DICOM) data from the CBCT consist of voxels demonstrating their own gray level based on specific calculations of absorbed X-ray. A voxel is the equivalent of a 2D image pixel with an added third dimension for depth. The computer uses an algorithm for voxel data to reconstruct 3D volume. Following, a 3D surface model of the bone can be generated using a specific algorithm, which is a process qualified of mesh generation [19]. To identify the surface of interest, a threshold value for the grey scale corresponding to the bone has to be specified by the operator in order to make voxels visible or not [20]. Special 3D reconstruction software (Mimics, Leuven, Materialise, Belgium) was used to perform these procedures. The surface of the bony structure to be grafted was saved as a standard tessellation (.STL) file. Data were transferred to a 3D printer and a solid model was constructed using autoclavable nylon polyamide (3D Neovision, Lyon, France). The 3D printing technology used for physical reconstruction was either stereolithography or fused-deposition modeling, which have both shown adequate sufficient precision for the surgical purposes described in this paper. Additional information describing these technologies have been reported elsewhere [19].

### Graft material

**Fig. 2:**
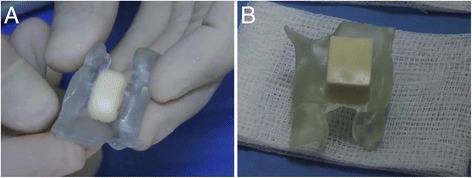
A corticocancellous block was manually milled to fit into the defect on a 3D model for greater stability. **a** The block before manual milling. **b** The milled block

In order to fit into the defect while maintain stability, a 15 mm long, 10 mm wide and 5 mm thick cube of freeze-dried corticocancellous block (TBF, Mions, France) was manually milled over the 3D printed model of the maxillary bone. At this stage, two aspects are important to consider: accurate fit of the host block to minimize the remaining void between crestal bone and the graft, and rounding the sharp edge of the outer part of the block so as to not injure the covering flap during the healing period (Fig. 2a, b).

### Surgical procedure

Patients were instructed to take: 1) 2 g of amoxicillin 1 h before surgery (patients with an allergy to penicillin took 600 mg of clindamycin); 2) 60 mg of a steroidal anti-inflammatory drug for 4 days, starting the day of surgery; 3) 1000 mg of paracetamol (recommended for pain), which patients could take as needed but not more than 4 g per day; 4) mouthwash based on chlorhexidine digluconate 0.12% solution (daily for 1 week).

Patients were anesthetized using local infiltration of 1.7 mL of articaine (60.277 mg) with adrenaline (1/100,000).

**Fig. 3:**
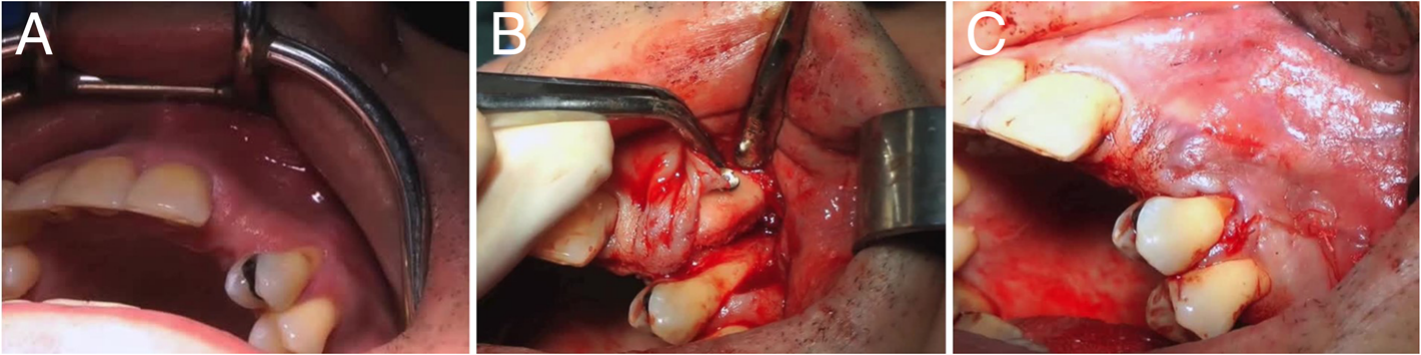
The allograft block is placed in the defect area through the tunnel and stabilized with a screw so that the screw head perfectly fits the cortical plate of the block. **a** Site before intervention. **b** Bone block with screw. **c** Sutures

A vertical releasing incision distal to the adjacent tooth was made on the buccal aspect starting from the collar in the keratinized tissue. Neither a crestal incision nor an incision in the sulcus was performed. A full-thickness mucoperiosteal tunnel flap was made using a periosteal elevator while being extremely careful not to tear the periosteum. The buccal and crestal aspects of the alveolar ridge were then exposed using subperiosteal dissection to allow both mobilization of the flap and primary closure without placing tension on the soft tissue following graft placement. Multiple perforations through the cortical plate were made using piezosurgery to ensure blood diffusion between the grafted bone and marrow cavity [21]. The allograft block was then placed in the defect area through the tunnel and stabilized with a screw so that the screw head perfectly fit the cortical plate of the block (Fig. 3a–c). Despite extreme caution during the milling stage to fit the graft, the presence of a void was possible, which could then be filled with particulate material recovered from the initial allogeneic block. No covering membrane was used. Soft tissue closure was achieved using 4.0 sutures. Sutures were removed 10–15 days following surgery during the first follow-up visit.

The optimal solution would be to not use a provisional prosthesis during the 6 month healing period. In the esthetic area, fixed temporary bridges were delivered to the patient, or they were given a removal prosthesis, which was designed so as to not impinge the graft.

During the healing period, patients were advised to pay attention to any potential soft tissue dehiscence, whereby a follow-up visit would be necessary.

**Fig. 4:**
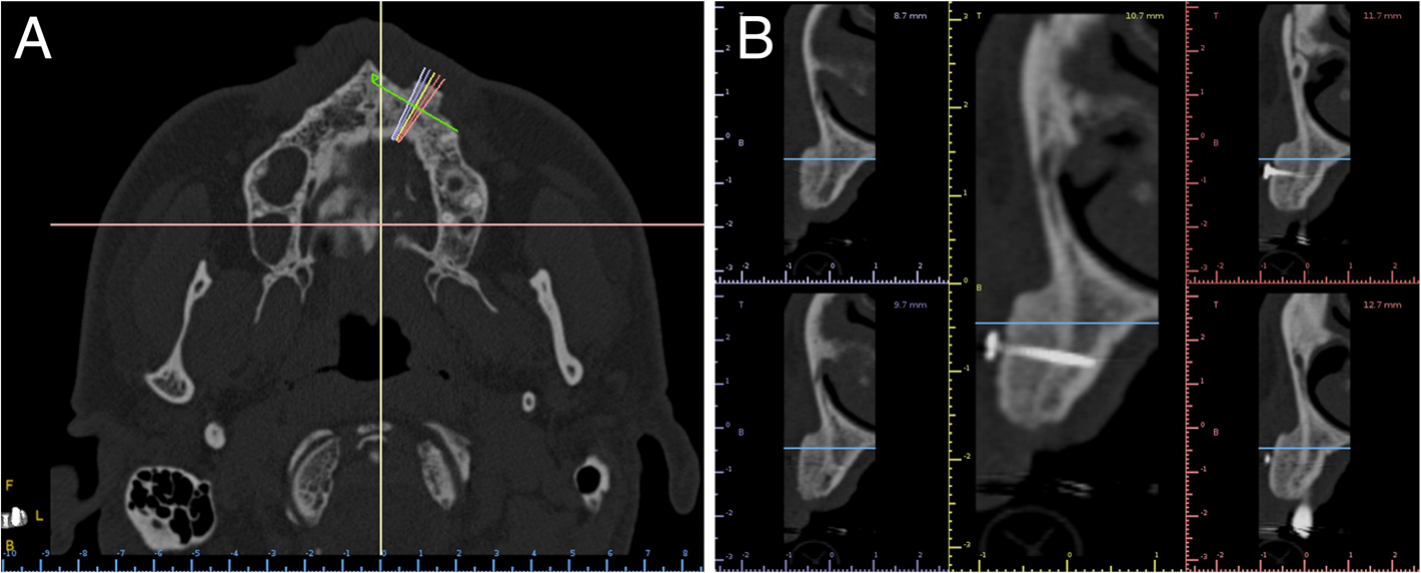
CBCT were obtain after 6 months to determine implant width and length, on axial (**a**) and cross-sectional (**b**) images

**Fig. 5:**
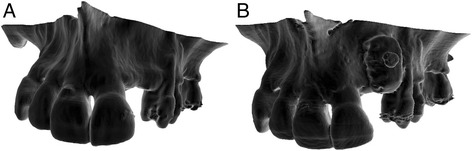
Photorealistic renderings of the maxilla. **a** Preoperative anatomic situation. **b** Anatomic situation 6 months after surgery

**Fig. 6:**
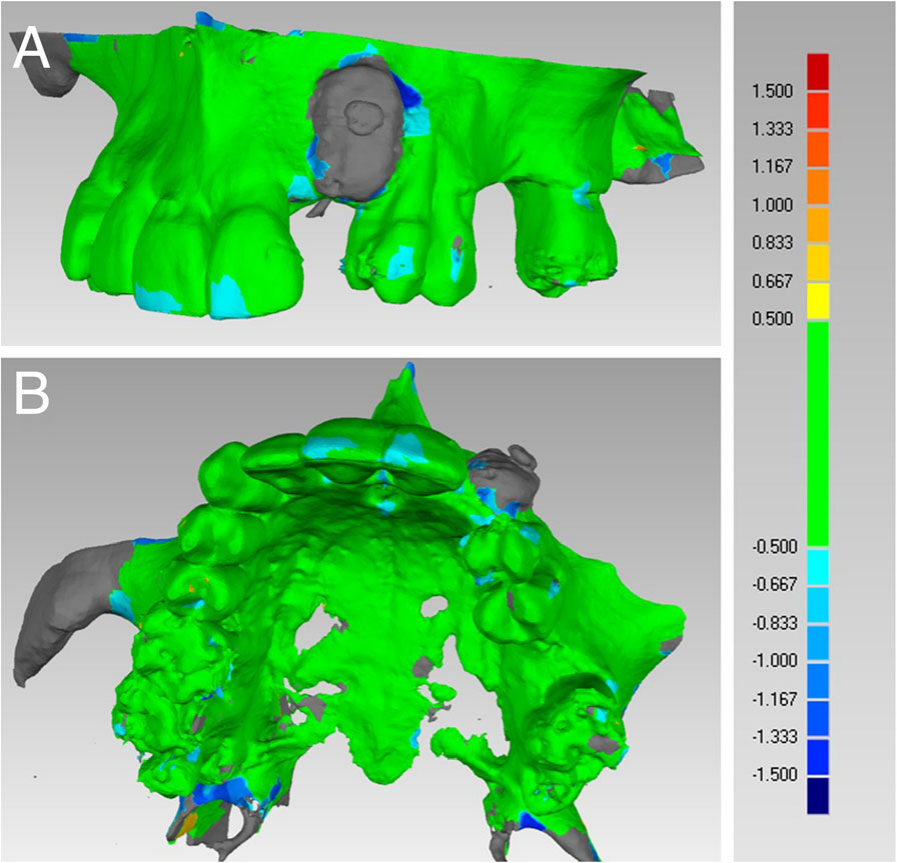
Superimposition of the preoperative maxillary 3D model and the postoperative 6-month control model, by means of a reverse engineering software, for quantitative evaluation of volume gain. **a** Lateral view. **b** Occlusal view

Control CBCT scans were taken 6 months following surgery to determine optimal implant width and length (Fig. 4a, b). DICOM files obtained from CBCT were imported into a 3D reconstruction software program (Mimics, Materialise, Leuven, Belgium) where after accurate segmentation, a 3D model of the postoperative condition was obtained and saved as an. STL file. Photorealistic renderings were obtained for both pre- and post-operative maxillary 3D bone models in order to evaluate gain in terms of volume (Fig. 5a, b). The post-operative 3D bone model was then superimposed to the pre-operative bone model via powerful reverse engineering software (Studio 2012 Geomagics, Morrisville, NC, USA). In order to quantitatively evaluate volume gain, a colorimetric map was used to evaluate distances between the two models (Fig. 6a, b).

**Fig. 7:**
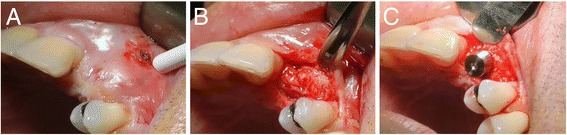
6 months after the reconstructive surgery, the implant was placed in position. **a** Site before intervention. **b** Surgical flap is raised. **c** The implant is placed

Following 6 months of recovery, a second-stage surgery for implant placement was performed. Access to the augmented ridge was obtained via a midcrestal incision. The fixation screw was removed through the gingiva via a small linear incision or after flap reflexion. If the graft was stable and augmented bone was strong enough to place the implant in the appropriate position, the implant was placed and exposed for impression following a 4 month recovery period (Fig. 7a–c).

## Results

From May 2013 to February 2014, six patients were treated, and 11 blocks were used for the placement of 12 implants (Table 1). During surgery, all scaffolds closely matched the shape of the defects: this reduced the operation time and contributed to good healing. No intraoperative complication occurred in this study; moreover, there were no adverse events (such as inflammation, dehiscences or flap re-opening) during the recovery process. At the re-opening stage, the 3D reconstruction and the resorption rate of the scaffolds were checked. Patient number 1 had a space between the screw head and the cortical plate of the bone block, which was marked. This was likely attributable to the uncontrolled movements of the removable provisional prosthesis. No other patients demonstrated resorption with the screw head remaining on the cortical plate of the bone block. In all patients except one, planned implants were placed 6 months following grafting (in one patient, fixture placement was postponed because the patient moved to another city for a period). All implants received the planned prosthesis 4 months later. All patients attended follow-up visits following the recovery period. Radiographic assessments did not demonstrate the presence of any adverse events or complications.

**Table 1:** Details of the treated patients

Patient no.	Number of blocks	Location in the maxilla	Number of implants	Type of prosthesis	Adverse events
1	4	Anterior	5	Complete fixed restoration	Resorption
2	1	Anterior	1	Single tooth	No
3	2	Anterior	2	Single tooth	No
4	1	Anterior	Not yet		No
5	1	Anterior	1	Single tooth	No
6	2	Anterior	3	Partial fixed restoration	No
Total	11		12		

## Discussion

The present technique for the fabrication of customized allogeneic bone grafts for maxillary ridge reconstruction can provide different benefits for the patient and for the clinician. In fact, the accurate reproduction of the patient’s anatomy helps to simplify the surgery, to dramatically reduce the time needed for the surgical procedure and therefore the morbidity and risk of infection for the patient [16–18]. In addition, the increased stability of the allogeneic bone block may contribute to faster and better bone healing and graft incorporation/consolidation [14, 15]. In our present study, no gaps were evidenced between the custom-made synthetic scaffolds and the natural bone during the surgery.

In our present study, in addition, the customized allogeneic bone grafts were be adapted on the bone defects using a minimally invasive subperiosteal tunneling technique.

A previous study has assessed the effects of bone block grafting combined with a tunneling technique in animals [22], but this procedure has never been tested in humans. The procedure described in this paper accurately indexes each prepared allograft block to its corresponding defect site by attaching it directly to the 3D model with screws. This technique enhances adaptation of the preparation by providing a real 3D view of the defect and the possibility of turning around the connection area between the graft and the host site to eliminate any remaining void. This technique also makes it possible to round the outer edge of the block properly so as to not injure the covering flap. Moreover, it is noteworthy that use of a stereolithographic model as a template allows the surgeon to shape the graft without regard to hemostasis or time pressure, while also shortening time of the overall surgery. For this technique we used an allograft instead of an autologous block. The major limitation of autologous bone is the donor site including potential risk of morbidity at the site, impaired tactility and sensitivity and apical pathology [23].

Irrespective of applied graft materials, the tunnel technique is attractive since it involves a minimally invasive procedure associated with a more conservative surgical entry, while also shortening surgical time and lowering postoperative morbidity (namely flap re-opening, pain and edema). Clinical outcomes observed in this case series are consistent with previous studies that have demonstrated the sub-periosteal tunneling procedure prevents graft exposure with minor postoperative complications [24, 25].

Minimal graft resorption could be observed at implant placement following the 6 month recovery period. Similar results have been reported by Nissan et al. [21] in 2011, demonstrating a resorption of 0.5 ± 0.5 mm with a similar healing period. In the 25 cases reported on by Nissan et al. [21], bone blocks were covered with bovine bone particulate mineral or mineralized freeze-dried allograft bone and covered with resorbable collagen membrane. They suggest that minimal resorption is attributable to coverage with the particulate bone and collagen membrane. As such, in the present case series, we obtained similar results without particulate material and membrane. Differences in observations may be related to use of cortico-cancellous and not only cancellous bone block in the present case series, which resulted in a more resistant graft. Indeed, cortical fraction provides adequate rigidity to withstand tension from the overlying soft tissues and/or functional pressure and is less susceptible to resorption [26, 27].

Rigid fixation of the block due to both screw and periosteum pressure is probably one factor contributing to success. Hurzeler et al. [28] have demonstrated that small movement during the early stages of recovery promoted differentiation of mesenchymal cells into fibroblasts instead of osteoblasts.

Minimally invasive techniques are generally considered to lead to better results in reconstruction processes given the importance of tissue injury influences speed and quality of healing [29].

The integrity of the periosteum could be one key factor for minimal resorption. This could be expected to improve re-ossification of the reconstructive material based on osteogenic cell penetration and adhesion and neovascularization useful for supplying individual cells with nutrients and oxygen. Indeed, the vascularization process continues over time in the host tissue from the outer area to the volume core. As a consequence, cells located at the core of the graft die faster due to ischemia in the central part, which can result in incomplete colonization [30, 31] that is limited to the external or other layer of the scaffold. Thus, time required for development of the vascular network to the entire graft volume depends on both grafted bone volume and integrity of the periosteum.

The role of the periosteum in osteogenesis and providing mesenchymal cells and osteoblasts is well documented [32–35]. This has been confirmed by Xuan et al. [22], who demonstrated a marked difference in new bone formation when placing a bone block through a tunnel with re-ossification at the base of the block and in the more coronal regions of the graft. In contrast, for the conventional flap procedure, new bone is generally limited to the base of the block.

A potential advantage of our present procedure could be the possibility of using a larger bone block with potentially minimal resorption. This can have a high impact on the long-term success of the implant since marginal bone loss around the implant is related to bone thickness [36]. More investigations are needed to confirm this hypothesis because bone volume augmentation is limited by lifting the periosteum to avoid perforation.

Extra cost due to fabrication of the 3D solid model may be a limitation, but it is offset by shortened surgical time, elimination of the covering particulate and the membrane given similar results obtained in this case series. Moreover, the evolution of accurate low-cost printers accessible for a reasonable investment could represent a favorable solution, even in a dental office.

Milling the block using a milling machine has also been suggested [37–40]. This approach can be useful when a large block with a complex shape is needed for an extensive defect. However, the combination with the tunnel technique can be limited by the size of the vertical releasing incision and laxity of the soft covering tissues. First and foremost, proper positioning of the graft and perfect wound closure must be ensured.

The surface model derived from the 3D CBCT based on the gray scale is open to variability [41]. The accuracy of segmentation is based on the gray-value of each voxel and depends on the threshold value selected by the operator. Thus, this process can differ from one operator to another and may result in loss of surface precision. To overcome those potential limitations, delegating this time-consuming stage to a 3D specialist may be a viable solution in a general clinical practice.

Difficulties in obtaining clear visibility of the site and perfect handling of the graft have been reported, regardless of anatomical considerations. Moreover, close attention must be paid to the perfect fitting of the graft to the recipient bed during the screw positioning stage in order to avoid rotations and misplacement. A learning period is mandatory to achieve adequate and reproducible results.

Our present clinical studies has limits, such as the limited number of patients treated and surgeries performed, as well as the short follow-up time. Moreover, the present technique requires the allogeneic bone blocks to be manually adapted over the jawbone 3D replicas, and this can be considered a limitation of the present study; today, in fact, it is possible to mill custom-made scaffolds using milling machines, with a simplification of the procedures, a reduction of the manual part for the clinician and most of all, a potentially better accuracy [37–40]. In addition, in the present study, an histologic/ histomorphometric evaluation of the integration of the allogeneic bone blocks is missing; therefore, in the next studies it would be advisable to retrieve bone samples during implant bed preparation, in order to verify the percentage of actually regenerated bone, and that of residual allogeneic bone. Finally, it is important to point out that allografts carry some inherent limitations, such as delayed vascular penetration, slow bone formation, higher rates of bone resorption, and the possibility of immunogenicity and disease transmission [42]. About this last aspect, improved screening, testing, and processing techniques have significantly reduced the risk of infection through bone allografts [42]. However, total sterility is not a practically attainable concept with any human tissue, and in the next future, it is likely that allografts will be replaced by synthetic materials [42].

## Conclusions

In the present study, eleven onlay customized allogeneic bone grafts were prepared and implanted in 6 patients, using a minimally invasive subperiosteal tunneling technique. The scaffolds closely matched the shape of the defects: this reduced the operation time and contributed to good healing. No complications were reported during the healing period, even if one patient experienced bone resorption of the graft, due to mobilization of the customized bone block. Six months after bone regeneration, the patients received their implants and 4 months later, their implant-supported restorations. These observations suggest that customized bone allografts can be successfully used for horizontal ridge reconstruction of the anterior maxilla, with reduced patient morbidity and surgical time. However, further studies on a larger sample of patients, with a longer follow-up and possibly an histologic evaluation are needed to confirm the present observations.

## Abbreviations

2D: Two-dimensional
3D: Three-dimensional
CBCT: Cone beam computed tomography
DICOM: Digital imaging and communication in medicine
STL file: Standard tessellation
